# Metabolism of no-carrier-added 2-[^18^F]fluoro-L-tyrosine in rats

**DOI:** 10.1186/1756-6649-8-4

**Published:** 2008-11-07

**Authors:** Joël J Aerts, Alain R Plenevaux, Christian F Lemaire, Fabrice Giacomelli, Geoffrey I Warnock, Christophe L Phillips, André J Luxen

**Affiliations:** 1 Centre de Recherches du Cyclotron, Université de Liège, Liège, Belgique

## Abstract

**Background:**

Several fluorine-18 labelled fluoroamino acids have been evaluated as tracers for the quantitative assessment of cerebral protein synthesis *in vivo *by positron emission tomography (PET). Among these, 2-[^18^F]fluoro-L-tyrosine (2-[^18^F]Tyr) has been studied in mice at a low specific activity. Its incorporation into proteins is fast and metabolism via other pathways is limited. The present *in vivo *study was carried out in normal awake rats using no-carrier-added 2-[^18^F]Tyr. Under normal physiological conditions, we have studied the incorporation into proteins and the metabolism of the tracer in different brain areas.

**Methods:**

No-carrier-added 2-[^18^F]Tyr was administered to awake rats equipped with chronic arterial and venous catheters. The time course of the plasma activity was studied by arterial blood sampling. The biodistribution of the activity in the main organs was studied at the end of the experiment. The distribution of radioactive species in plasma and brain regions was studied by acidic precipitation of the proteins and HPLC analysis of the supernatant.

**Results:**

The absolute uptake of radioactivity in brain regions was homogenous. In awake rats, no-carrier-added 2-[^18^F]Tyr exhibits a fast and almost quantitative incorporation into the proteins fractions of cerebellum and cortex. In striatum, this incorporation into proteins and the unchanged fraction of the tracer detected by HPLC could be lower than in other brain regions.

**Conclusion:**

This study confirms the potential of 2-[^18^F]fluoro-L-tyrosine as a tracer for the assessment of the rate of protein synthesis by positron emission tomography. The observed metabolism suggests a need for a correction for the appearance of metabolites, at least in plasma.

## Background

For years, amino acids, the basic units of proteins, have been identified as a potential tool for the study of the *in vivo *cellular kinetics of protein synthesis [1]. In order to achieve this in humans, implementing nuclear medicine applications, researchers must make use of either radioactive forms of natural amino acids or radioactive analogues which can be incorporated into proteins.

Although radioactive iodinated derivatives have been studied for single photon emission computed tomography (SPECT) and are still of particular interest today [2], compounds labelled with positron emitters, suitable for the positron emission tomography (PET), were the main subjects of study in human functional imaging for the last twenty years. This relies on the high sensitivity and the quantitative capability of PET [3] and on the characteristics of carbon-11, an isotope of a naturally existing element in biological molecule, or fluorine-18, a bioisostere of naturally existing chemical groups, for example the OH group [4,5].

L-[S-methyl-^11^C]-methionine has been used to generate interesting clinical results for the diagnosis of brain tumours [6]. Unfortunately, due to the very short half-life of carbon-11 (20.4 minutes), the use of tracers labelled with this radionuclide is logistically only possible on sites equipped with their own cyclotron. Even in this case, the completion of a high number of patient examinations with the same radiopharmaceutical batch is technically difficult except if several PET scanners are simultaneously accessible. This physical limitation therefore restricts the usefulness of carbon-11 labelled tracers in the study of biochemical processes to a time scale of maximum one hour.

With a half-life of 109.8 minutes, fluorine-18 presents better characteristics for the geographic expansion of radiopharmaceuticals supply. This capability has been practically proven worldwide with the use of the PET tracer 2-[^18^F]fluoro-deoxyglucose ([^18^F]FDG). Moreover, the longer half-life of fluorine-18 is more suitable for the study of processes with a time scale of more than one hour, such as brain protein synthesis by example.

**Figure 1:**
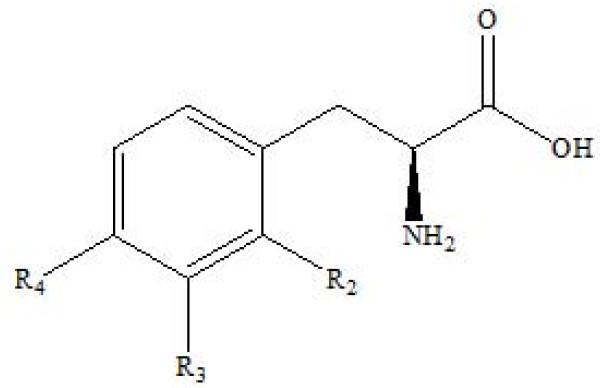
**Structure of fluorinated aromatic amino acids**. 2-fluoro-L-phenylalanine (R2 = F, R3 = R4 = H); 3-fluoro-L-phenylalanine (R3 = F, R2 = R4 = H); 2-fluoro-L-tyrosine (R2 = F, R3 = H, R4 = OH), 3-fluoro-L-tyrosine (R3 = F, R2 = H, R4 = OH); 4-fluoro-L-phenylalanine (R2 = R3 = H, R4 = F).

The fluorinated aromatic amino acids 2-, 3- and 4- fluorophenylalanine, 2- and 3- fluorotyrosine (figure 1) have been described by geneticists as potential surrogates for canonical phenylalanine and tyrosine in an expanded repertoire of amino acids corresponding to a less restricted genetic code [7]. All of them have been previously been evaluated as [^18^F] labelled tracers for the quantitative assessment of cerebral protein synthesis *in vivo *[8]. 2-[^18^F]fluorophenylalanine and 2-[^18^F]fluorotyrosine have also been considered for application in tumour imaging [9].

Among the few fluorinated amino acids incorporated into proteins, 2-[^18^F]fluoro-L-tyrosine (2-[^18^F]Tyr) is the most promising. Its incorporation has been studied in mice at a low specific activity (10–20 GBq/mmol). This revealed a very simple metabolism as well as rapid incorporation into proteins [10].

The present *in vivo *study was carried out in awake rats using no-carrier-added (n-c-a) 2-[^18^F]Tyr, under normal physiological conditions. The incorporation of this tracer into proteins and its metabolism were studied. The use of a tracer with higher specific activity increases the sensitivity for the detection of potential biochemical mechanisms, in which the fluorinated analogue could specifically be involved, in comparison to the natural circulating amino acid.

## Methods

3-O-methyl-6-fluorodopa (3-OMFD), 3,4-dihydroxy-6-fluorophenylacetic acid (FDOPAC), 6-fluorodopamine (FDA) and 6-fluorohomovanillic acid (FHVA) were synthesized following previously published procedures [11]. 2-fluoro-L-tyrosine and 6-fluoro-L-dopa were purchased from SanverTech. The n-c-a 2-[^18^F]Tyr used in this work was obtained through two different enantioselective methods of synthesis previously published by Lemaire [12,13]. The first was described for 6-[^18^F]fluoro-L-dopa and adapted for 2-[^18^F]fluoro-L-tyrosine from 4-methoxy-2- trimethylammoniumbenzaldehyde triflate and (2S)-1-tert-boc-2-tert-butyl-3-methyl-4-imidazolidinone. The second uses catalytic phase-transfer alkylation with a chiral quaternary ammonium salt derived from a Cinchona alkaloid. The enantiomeric purity and the specific activity were controlled at the end of synthesis and were above 95% and 37 GBq/μmol respectively.

All animal experiments were approved by the Ethical Committee at our institution. Chronic cannulations of rats (male, Sprague Dawley, 180–250 g) were performed in the abdominal aorta and in the vena cava under general anaesthesia (ketamine 35 mg/kg and medetomidin 0.35 mg/kg) following the procedure described by Lestage [14]. The animals were allowed to recover for a minimum of 4 days before being used for metabolism studies. Most of them were injected with the tracer within the first week after surgery. Some of them were kept up to 4 weeks before injection. Twenty hours before the injection of the tracer, the food was removed from the cage. The arterial line was used for blood sampling and the other for i.v. injection of the tracer. The two lines were flushed with saline a few minutes before use in order to check potency. 14 rats (230–410 g) were injected with 20–325 MBq of n-c-a 2-[^18^F]Tyr. The tracer was delivered as a bolus of maximum volume 0.75 ml in 15 s. Samples of blood were obtained via the arterial cannula during the first minutes after the injection and at different times (15, 30, 45, 60 and/or 90 minutes) thereafter. The line was flushed with saline between each sample to avoid any loss of potency and to ensure the homogeneity of the next sample. The animals (n = number of rats) were sacrificed after 30 (n = 2), 60 (n = 5), 90 (n = 3) or 120 (n = 4) minutes. After the final time point, samples of blood were taken and the major organs were dissected. The brain was dissected to obtain striatum, cortex and cerebellum.

Trichloroacetic acid (TCA) was used to precipitate the proteins from biological samples in order to assay the radioactive content associated with proteins and to perform a chromatographic analysis of the radioactive compounds in solution in the supernatant after centrifugation. The solution used for the precipitation was Na_2_S_2_O_5 _100 mg, NaEDTA.2H_2_O 10 mg, TCA 20 g, dissolved in water up to 100 ml.

Radioactivity was measured in weighed fractions of plasma. For intermediate and final plasma samples, proteins were precipitated with TCA solution and separated by centrifugation (5°C, 5 minutes, 8000 RPM) before counting. The supernatant was analysed by HPLC. Radioactivity was measured in the organ samples and in one half of the three dissected parts of the brain. The second halves were sonicated with water (Sonics & Materials inc., Vibracell VXC-400). The proteins were precipitated with TCA solution and separated by centrifugation (5°C, 5 minutes, 8000 RPM) before counting. The supernatant was analysed by HPLC.

The metabolites analysis was performed by HPLC [11] on fractions of the supernatants obtained after precipitation and centrifugation. Column: Phenomenex Bondclone 10 μm C18 300 mm × 3.9 mm. Injection volume: 100 μl. Mobile phase: 80% of 0.1 M NaH_2_PO_4_, 0.1 mM EDTA, 2.6 mM octane sulfonic acid sodium salt and 20% methanol, pH 3.1. The flow rate was 1 ml/min from start to 8 minutes and was then increased in 30 seconds interval at 1.5 ml/min. Fractions of 30 seconds were collected for 16 minutes. Capacity factors: FDOPA: 0.8; 3-OMFD: 1.5; FTYR: 1.9; FDOPAC: 2.6; FDA: 3.3; FHVA: 5.0 (k' = t_r_-t_0 _/t_0_, t_r_: retention time of the solute, t_0_: column dead time).

Radioactivity measurements of the biological samples and of the fractions collected during HPLC were performed with a NaI counter (Cobra II autogamma, Packard).

## Results

### Biodistribution

The biodistribution of n-c-a 2-[^18^F]Tyr after injection, expressed as the percentage of the injected activity recovered per gram of tissue (%IA/g) at different times is presented in table 1 [see Additional file 1]. The values observed for different regions of the brain (cerebellum, striatum and cortex) are presented in table 2 [see Additional file 2], column "total". Most of the tissues underwent a rapid uptake of activity without major evolution after 30 minutes. In particular, brain and brain sub-regions had very similar and stable uptakes. In plasma and bone uptake increased with time.

### Metabolism of the tracer

In the radioactive pool observed in the different tissues, the distribution of the radioactive species was studied by precipitation with TCA (precipitated fraction, proteins) and by HPLC analysis of the supernatant (unchanged tracer fraction). The %IA/g recovered in the precipitated fraction was calculated from the counts in these fractions. The percentage of unchanged tracer in the fractions collected by HPLC was calculated from the counts in these fractions and used to calculate the percentage of the injected activity recovered unchanged per gram of tissue in the supernatant fraction. This distribution of radioactive species is presented at different times for plasma, cerebellum, striatum and cortex (table 2 [see Additional file 2]). For plasma, table 3 [see Additional file 3] includes the measured percentages of activity associated with proteins in the total activity and with the unchanged fraction in supernatant obtained after proteins precipitation. This table also includes the calculated values for the unchanged fraction in the total activity and the remainder (100 – % of proteins in total- % of unchanged in total). Although the numbers of observations are small in most time points, some tendencies can be qualitatively extrapolated from these data. The incorporation into proteins was the most important metabolism observed for the tracer. This incorporation was rapid and reached high values already at 60 minutes. The transformation of the tracer into the catecholamine derivatives was a secondary process with very low quantitative importance. When comparing in table 2 [see Additional file 2] the values of the % IA/g associated with proteins for cerebellum and striatum, the data suggest that the incorporation seemed to be less important for striatum.

### Input function

**Figure 2:**
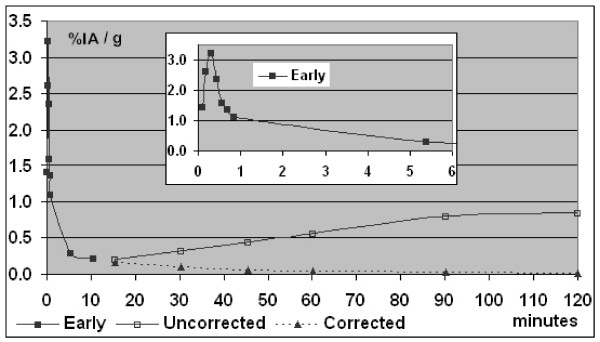
**Input function**. Kinetics of total arterial plasma activity with and without correction for appearance of metabolites. Values for times less than 15 minutes are uncorrected measured values for one rat (early, full squares). Open squares represent the average of all measures of uncorrected %IA/g of plasma. Full triangles represent the average values corrected for the appearance of metabolites using percentages reported in table 3. Insert: zoom on early times.

The average values of plasma activity over time were corrected for the global appearance of metabolites (TCA precipitated and soluble) with the values of percentages of the total activity recovered unchanged (table 3 [see Additional file 3]) to obtain the kinetics of total arterial plasma activity after injection of n-c-a 2-[^18^F]Tyr (figure 2). In this figure, the points for times before 15 minutes represent the observed values for one rat only and are not corrected. This part of the figure shows an example plasma profile of total activity after the intravenous injection. From 15 minutes, all values are the average of n observations (table 3 [see Additional file 3]) with or without correction for the transformation of the tracer. The plasma input function obtained clearly shows an increasing contribution of the protein pool over time, after the initial peak of injection.

## Discussion

During the last years, fluorinated tracers [15], especially amino acids [9], were mainly studied for their application in tumour imaging. A major aim of these developments is to improve, in comparison with the routine [^18^F]FDG scan, the specificity for tumours and the contrast with the surrounding tissues, especially in brain cancers. For this purpose, nuclear imaging is based on the ability of the tumour cells to concentrate amino acids more than normal tissues as a result of an increased efficiency of the transport through the cellular membrane by active transport systems. Moreover, the increased proliferation rate in tumours goes together with a higher protein synthesis rate (PSR). Thus, for some labelled amino acids, characterised by their capability to be incorporated into proteins, increased transport is coupled to a subsequent incorporation step. The two steps are kinetically different. Transport is a relatively rapid process allowing imaging only 20 minutes after injection. Incorporation into proteins is a slower process that requires imaging at longer times post-injection. This represents an advantage in the use of fluorine-18 rather than carbon-11 for PET evaluation of PSR.

Different parameters, including amino acid transport and PSR, are needed to fully characterise a tumour and monitor the response to treatments [9]. An incorporated labelled amino acid has the advantage of potentially imaging, with a single injection, either early transport of amino acids into cells and the delayed incorporation into the proteins afterwards. Nevertheless, this may only be a virtual advantage for tumour imaging as some studies indicate that the major phenomenon leading to an increased uptake of amino acids in tumors is an enhancement of amino acid transport rather than an increased PSR. So, the uptake of L-[S-methyl-^11^C]methionine in mice brain was not influenced by the use of a inhibitor for protein synthesis [16]. In the same conditions, the uptake of 2-[^18^F]Tyr was partially reduced. In another study evaluating 2-[^18^F]Tyr in patients with gliomas [17], pharmacokinetic modelling of the results indicated that the main difference between normal and tumorous tissues was a significantly increased transport rate constant from plasma to tissue. Thus, these two studies suggested that 2-[^18^F]Tyr could be used to assess the amino acid transport system. In this context, more recent developments have led to the evaluation of O-(2-[^18^F]Fluoroethyl)-L-tyrosine. This tracer, not incorporated into proteins, has the important advantage of being available with a relatively high radiochemical yield (40%) allowing the distribution of the product and its use to a larger extent. As a result of this availability, it has already been evaluated for different applications in clinical oncology [18]. In comparison, 2-[^18^F]Tyr suffers from a less efficient radiosynthesis and must thus still be better evaluated. In a small study for whole-body tumour imaging, the tracer was less sensitive than [^18^F]FDG for staging NSCLCs and lymphomas [19]. In another study focusing on primary brain tumors, 2-[^18^F]Tyr allowed a better tumor to background contrast in low-grade gliomas [20].

In addition to neuroendocrine activity, nucleic acid synthesis and gene transcription, protein synthesis has been described as a potential basic mechanism for brain synaptic plasticity [21]. Thus, incorporated amino acids may have further advantages for the study of various non tumourous processes. For example, the evaluation of PSR during brain development, sleep or memory consolidation is only possible with an incorporated tracer. Thus, beside uses in oncology, imaging PSR with amino acids presents a real interest for functional research, as well as potential applications in pathologies related to neurological physiological processes. The use of a single pharmaceutical agent adapted for several applications is a good approach from an economical point of view. As markedly demonstrated by [^18^F]FDG, this is especially true for PET pharmaceuticals, whose constraints concerning logistics, radioprotection and quality assurance, are considerable. All these arguments are in favour of the development of a multi-purpose [^18^F] fluorinated incorporated amino acid.

2-[^18^F]Tyr was identified early by Coenen and colleagues [10] as a promising tracer for PSR assessment in an extensive study in mice. At a low specific activity, its acceptance by the amino acid-tRNA synthetase has been proven by phenolic extraction of the tRNA bound fraction. The rapid and general incorporation into proteins has been confirmed by SDS gel electrophoresis. A very small proportion of metabolites was detected in striatum tissue and the authors raised the possibility of a very small entrance of the tracer to the catecholamine metabolic pathway [22].

The present study was performed using n-c-a 2-[^18^F]fluoro-L-tyrosine allowing the injection of a high amount of radioactivity (up to 325 MBq) supported by a low amount of non radioactive equivalent (less than 10^-2 ^μmole). The higher specific activity used in this study may not be of major physiological consequence since the concentration of competing amino acids in the plasma is high under fasting conditions. On the other hand, one can not exclude the existence of a biochemical mechanism (e.g. transport or metabolism) truly specific for the fluorinated analogue, in which the non fluorinated natural amino acid could not be involved at all. Using a higher specific activity increases the sensitivity for the detection of these potential mechanisms. The results of the present study can thus be interestingly compared to the results published for mice [10].

From the biodistribution data presented in table 1 [see Additional file 1], we can observe that the fluorinated analogue rapidly passes the blood-brain-barrier as the uptake in brain already reached a high value at only 30 minutes post injection. The absolute uptake into rat brain tissue (~0.15%IA/g) was lower than the value reported for mice (~1.3%IA/g at 40 min.; ~2%IA/g at 60 min.), as may be expected due to species differences (including size, weight and distribution volume). On the other hand, the total radioactivity of the plasma increased with time after the initial peak of injection, indicating the elimination of radioactive compounds in the blood. The uptake of radioactivity in bone increased with time and to a greater degree than in other organs such as muscle, heart or lung, indicating the probable liberation of [^18^F]F^-^during the metabolism of the tracer. The comparison of uptake in the different brain regions shows a homogeneous distribution of activity (table 2 [see Additional file 2]).

In all studied tissues, the radioactivity associated with proteins increased from 30 minutes to 120 minutes (table 2 [see Additional file 2]). Expressed as the percentage of the total activity in the tissue, this fraction at 60 minutes in rat cerebellum and cortex (respectively 84% and 85%) matches the reported value for mouse cortex (84%). In rats, the incorporation in non dopaminergic rich regions reaches almost 90% of the total activity at 120 minutes. On the other hand, the observed incorporation of tracer in the striatum may be less than in other brain regions (66% at 60 min.). This tendency should be confirmed by a more extensive study including a higher number of subjects and allowing accurate statistical evaluation of the observations.

The percentage of unmetabolised 2-[^18^F]Tyr in the supernatant after protein precipitation decreased with time in all tissues, indicating that the tracer enters another pathway that simply the incorporation into proteins. This percentage of unchanged 2-[^18^F]Tyr was smaller in the striatum at all time points, indicating that a fraction probably enters the catecholamine metabolic chain. These observations are compatible with the affirmation that 2-[^18^F]Tyr is a substrate for the enzyme tyrosine hydroxylase. A strict identification and a precise quantification of the metabolites require an improved methodology to concentrate the solutes before HPLC analysis. Nevertheless, radioactive compounds corresponding to products eluted before 2-[^18^F]Tyr (as FDOPA does) and after (as FDA and FHVA do), were observed for the striatum.

In our laboratory, the enantioselective synthesis of n-c-a 2-[^18^F]Tyr is now routinely performed by chiral phase-transfer alkylation [13]. This synthesis proceeds via the preparation of a [^18^F] labelled electrophilic agent, followed by alkylation and hydrolysis before HPLC purification. The radiochemical yield averages 25% (decay-corrected, 100 minutes). Although very good enantiomeric excesses can be obtained, this method suffers from a long duration and some steps are difficult to automate (bromination with gaseous HBr for example). Thus, it is crucial that the method be further improved in order to permit higher yields through a completely automatable pathway, as is already the case for some other fluorinated amino acids, such as O-(2-[^18^F]Fluoroethyl)-L-tyrosine [18].

## Conclusion

This study of the metabolism of 2-[^18^F]fluoro-L-tyrosine in rats confirms that this tracer is rapidly and extensively incorporated into cerebral proteins and is therefore well suited to the assessment of PSR *in vivo *by PET. A correction for the appearance of metabolites is advised for quantitative interpretation of the collected data at least in plasma. An improvement in the radiosynthesis is necessary to make 2-[^18^F]Tyr widely available for its application in oncology and to envisage the extended use of this multi-purpose tracer.

## Competing interests

The authors declare that they have no competing interests.

## Authors' contributions

AJL synthesized the metabolites references and supervised the course of the study. CFL developed and synthesized the radiotracer. ARP and JJA performed the animal surgery and the animal study. FG helped for the HPLC development, GIW for the surgery and CP for statistics. All authors read and approved the final manuscript.

## Pre-publication history

The pre-publication history for this paper can be accessed here:



## Supplementary Material

Additional file 1 **Table 1 – Biodistribution.** Percentages of the injected activity per gram at different times in different tissues (mean ± standard deviation on n observations). Click here for file

Additional file 2 **Table 2 – Distribution of radioactive species in plasma and brain sub-regions.** Percentages of the injected activity per gram of tissue at different times associated with proteins (column 3) and with the unchanged fraction of the tracer in the supernatant after precipitation of the proteins (column 4), mean ± standard deviation on n observations. These percentages are compared with the total percentages of the injected activity per gram in the different tissues (column 2). Column 5: the remainder is calculated as (Total % IA/g – Proteins % IA/g – Unchanged % IA/g). Click here for file

Additional file 3 **Table 3 – Distribution of radioactive species in plasma.** Percentages of the activity at different times associated with proteins in the total plasmatic activity (column 2) and with the unchanged tracer in the supernatant (column 3), mean ± standard deviation on n observations. Column 4: calculated values for the unchanged fraction in the total activity. Column 5: the remainder is calculated as (100 – % proteins in total – % unchanged in total). Click here for file
