# Supplementary figures and images for: Metabolism of no-carrier-added 2-[18F]fluoro-L-tyrosine in rats
Source: BMC Med Phys. 2008 Nov 7;8:4. doi: 10.1186/1756-6649-8-4 (PMC2606674; doi:10.1186/1756-6649-8-4)

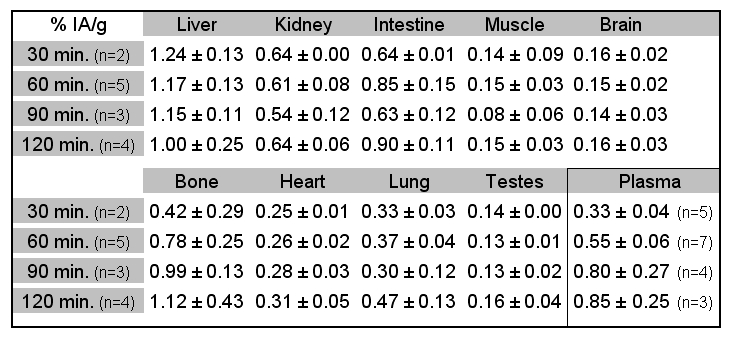

Supplement: Additional file 1 — Table 1 – Biodistribution. Percentages of the injected activity per gram at different times in different tissues (mean ± standard deviation on n observations). [file 1756-6649-8-4-S1.jpeg]

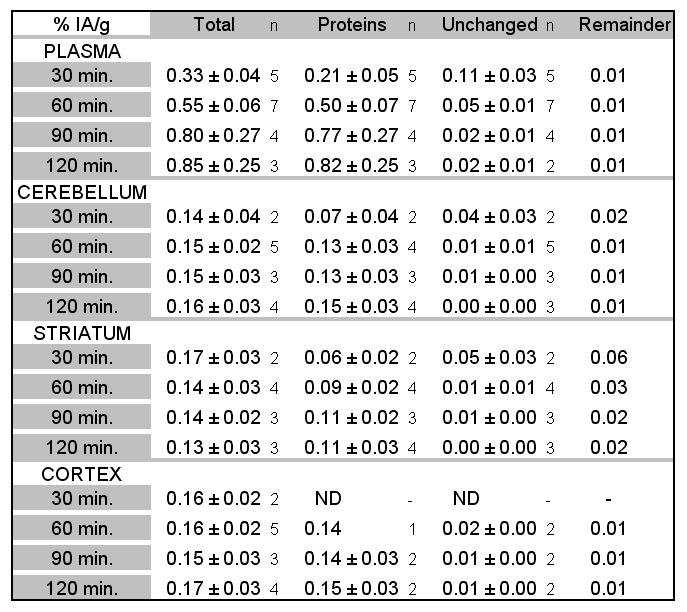

Supplement: Additional file 2 — Table 2 – Distribution of radioactive species in plasma and brain sub-regions. Percentages of the injected activity per gram of tissue at different times associated with proteins (column 3) and with the unchanged fraction of the tracer in the supernatant after precipitation of the proteins (column 4), mean ± standard deviation on n observations. These percentages are compared with the total percentages of the injected activity per gram in the different tissues (column 2). Column 5: the remainder is calculated as (Total % IA/g – Proteins % IA/g – Unchanged % IA/g). [file 1756-6649-8-4-S2.jpeg]

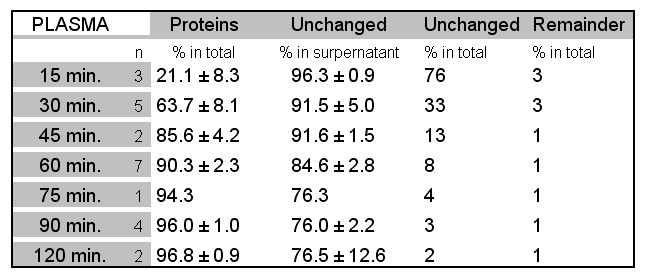

Supplement: Additional file 3 — Table 3 – Distribution of radioactive species in plasma. Percentages of the activity at different times associated with proteins in the total plasmatic activity (column 2) and with the unchanged tracer in the supernatant (column 3), mean ± standard deviation on n observations. Column 4: calculated values for the unchanged fraction in the total activity. Column 5: the remainder is calculated as (100 – % proteins in total – % unchanged in total). [file 1756-6649-8-4-S3.jpeg]
